# Directed DNA Shuffling of Retrovirus and Retrotransposon Integrase Protein Domains

**DOI:** 10.1371/journal.pone.0063957

**Published:** 2013-05-17

**Authors:** Xiaojie Qi, Edwin Vargas, Liza Larsen, Whitney Knapp, G. Wesley Hatfield, Richard Lathrop, Suzanne Sandmeyer

**Affiliations:** 1 Department of Biological Chemistry, School of Medicine, University of California Irvine, Irvine, California, United States of America; 2 Department of Computer Science, School of Information and Computer Sciences, University of California Irvine, Irvine, California, United States of America; 3 Institute for Genomics and Bioinformatics, University of California Irvine, Irvine, California, United States of America; 4 Department of Chemical Engineering and Materials Science, School of Engineering, University of California Irvine, Irvine, California, United States of America; 5 Department of Microbiology and Molecular Genetics, School of Medicine, University of California Irvine, Irvine, California, United States of America; 6 Department of Biomedical Engineering, School of Engineering, University of California Irvine, Irvine, California, United States of America; 7 CODA Genomics, Inc., Laguna Hills, California, United States of America; Universität Stuttgart, Germany

## Abstract

Chimeric proteins are used to study protein domain functions and to recombine protein domains for novel or optimal functions. We used a library of chimeric integrase proteins to study DNA integration specificity. The library was constructed using a directed shuffling method that we adapted from fusion PCR. This method easily and accurately shuffles multiple DNA gene sequences simultaneously at specific base-pair positions, such as protein domain boundaries. It produced all 27 properly-ordered combinations of the amino-terminal, catalytic core, and carboxyl-terminal domains of the integrase gene from human immunodeficiency virus, prototype foamy virus, and *Saccharomyces cerevisiae* retrotransposon Ty3. Retrotransposons can display dramatic position-specific integration specificity compared to retroviruses. The yeast retrotransposon Ty3 integrase interacts with RNA polymerase III transcription factors to target integration at the transcription initiation site. *In vitro* assays of the native and chimeric proteins showed that human immunodeficiency virus integrase was active with heterologous substrates, whereas prototype foamy virus and Ty3 integrases were not. This observation was consistent with a lower substrate specificity for human immunodeficiency virus integrase than for other retrovirus integrases. All eight chimeras containing the Ty3 integrase carboxyl-terminal domain, a candidate targeting domain, failed to target strand transfer in the presence of the targeting protein, suggesting that multiple domains of the Ty3 integrase cooperate in this function.

## Introduction

The yeast retrotransposon Ty3 integrates specifically at RNA polymerase III (Pol III) transcription initiation sites [1], but the Ty3 integrase (IN) is not well understood structurally. In contrast there is considerable information about the human immunodeficiency virus (HIV)-1 and prototype foamy virus (PFV) IN structures (reviewed in [2]), but the precise mechanism of their relatively subtle regional integration biases is not well understood [3]. Here, we used chimeric IN protein libraries to study integration DNA sequence specificity.

IN proteins of retroviruses and long terminal repeat (LTR) retrotransposons mediate integration of the replicated complementary (c)DNA into the host genome via nucleophilic attack by the cDNA 3′-OH at staggered phosphodiester bonds of the target DNA [4] (reviewed in [2], [5]). Structural and functional studies of retroviral IN proteins distinguish amino-terminal (NTD), catalytic core (CCD), and carboxyl-terminal (CTD) domains [2], [6], [7]. The NTD contains a conserved HHCC zinc-binding motif and has been implicated in multimerization. The CCD is the most conserved domain and contains the D,DX_35_E residues, which chelate metal cations required for catalysis. The CCD of HIV-1 IN is sufficient for reversal of integration (“disintegration”), but not for the forward reaction of strand transfer [8], [9]. IN shows specificity for cDNA ends and displays local target site sequence bias (reviewed in [2], [10]). Recent *in vitro* functional studies of PFV IN [11] coupled with the intact PFV IN crystal structure [11]–[13] have explicated the molecular basis of PFV IN binding to the cDNA ends. The intact PFV IN structure, together with previous partial structures of HIV-1 IN (e.g. [14] and reviewed in [2]), has also enabled more detailed modeling of the homologous HIV-1 IN [15]. Residues in the CCD of PFV IN interact in a base-specific pattern with the ends of the substrate cDNA. Residues of this domain also interact with the phosphodiester backbone of the target DNA, bending the target DNA at the point of nucleophilic attack. Interaction also occurs between target DNA and R329 and R362 in the CTD [12]. In addition retrovirus IN CTD has been demonstrated to have nonspecific DNA binding activity [5].

Specific substrate cDNA contacts and local target DNA sequence biases have been attributed to retrovirus IN. In addition, retroviruses display poorly understood but broad genomic targeting biases, which correlate in various ways with transcription patterns [16]. Based on the DNA binding activity demonstrated for retrovirus IN CTD and the general lack of CTD conservation, it has been speculated that the CTD contributes to long range targeting. However, currently the best understood host targeting factor is the chromatin associated protein, LEDGF, which is required for wild-type levels of HIV-1 integration *in vivo*. Despite the fact that LEDGF targeting appears to be specific for HIV-1, its interaction maps to the CCD with contributions from the NTD [17]–[20].

Retrotransposons differ from retroviruses in that many retrotransposons display dramatic genomic targeting [21]. Among fungal elements this is particularly apparent. Ty1 and Ty5, copia-like elements of *S. cerevisiae*, target the nucleosome-bound region within 750 bases upstream of RNA Pol III promoters [22], and Sir4, a heterochromatin component [23], respectively. Gypsy-like elements are classified based on the presence or absence of a chromodomain in the IN CTD [24]. In elements such as MAGGY, this chromodomain enables targeting to epigenetic modifications of histones [25] (reviewed in [25], [26]). Other gypsy-like elements target promoter regions. *Schizosaccharomyces pombe* Tf1 targets Pol II promoters [27], [28]. *S. cerevisiae* Ty3 targets Pol III initiation sites [1].

Thus, detailed structural information and *in vitro* assays are available for retrovirus IN proteins, but *in vivo* regional targeting is relatively subtle and complex. In contrast, retrotransposons can have dramatic targeting and discrete CTD subdomains have been implicated, but a recombinant *in vitro* integration reaction which recapitulates targeting has been lacking. Recent development of an *in vitro* assay in which Ty3 targeting is recapitulated by a single synthetic RNA Pol III transcription factor and recombinant Ty3 IN [29] motivated our current investigation of the domains required for Ty3 targeting to Pol III transcription initiation sites using a chimeric protein strategy.

We produced a chimeric IN protein library containing all twenty-seven properly ordered combinations of the NTD, CCD, and CTD domains of the IN gene from HIV, PFV, and Ty3. The protein domain boundaries of HIV and PFV were taken from X-ray crystal structures, while those of Ty3 were predicted based on molecular modeling and alignment with retrovirus integrases. The Ty3 IN CCD structure was modeled and aligned with PFV and HIV-1 IN CCDs to facilitate definition of the Ty3 CCD boundaries. A directed shuffling method adapted from fusion PCR was used to assemble the three full-length recoded native genes and 24 chimeric genes. We expressed and purified the 27 recombinant recoded native and chimeric IN proteins and assayed strand transfer for each of the three donor substrates.

## Materials and Methods

### 1. Domain Boundary Identification

**Figure 1:**
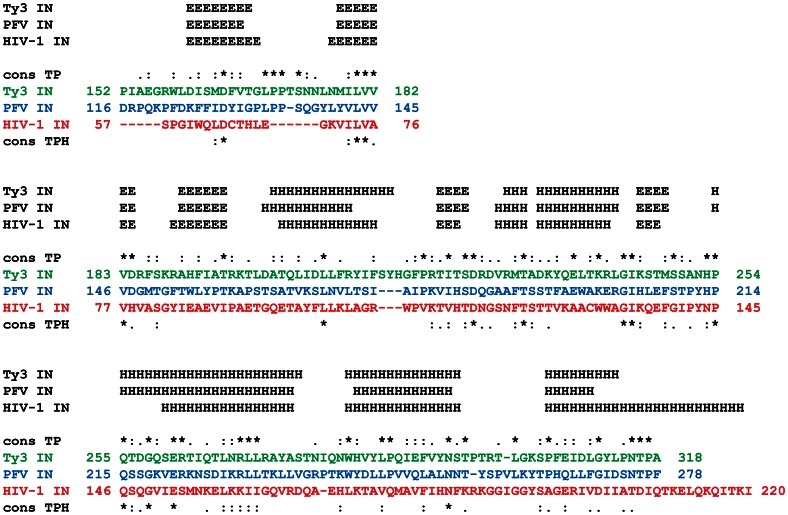
Alignment of CCD domain for Ty3, PFV, and HIV-1 IN proteins. PFV and HIV-1 sequence and secondary structure alignments were adapted from Hare, et al. [13] and Valkov, et al. [11]. Ty3 sequence and secondary structure alignments are the consensus of several methods as described in the text. “H” denotes both 3–10 and alpha helices. “E” denotes extended (beta strand). “cons TP” denotes conservation between Ty3 and PFV, and “cons TPH” conservation between Ty3, PFV, and HIV-1, using the ClustalW conservation groups [32]. Note that PFV sequence numbering is according to NCBI but the actual chimeric IN starts at the fourth residue in the PFV sequence. Conserved D,D X_35_E catalytic residues as numbered in the alignment are Ty3 IN: D164, D225, E 261; PFV: D128, D185, E221; HIV-1: D64, D116, E152.

HIV-1 and PFV IN [2] structural and *in vitro* studies have defined IN NTD, CCD, and CTD domains [6], [9], [13], [30]. Because comparable information is not available for Ty3 IN, candidate domain boundary assignments were predicted using comparative *in silico* approaches. We focused on the definition of the Ty3 CCD, using sequence alignment, evolutionary mapping, fold recognition, domain prediction, threading, and refinement. The CCD domain is the most similar to retroviral domains, and also delimits the flanking NTD and CTD domains. Initial candidate domain boundaries were obtained from multiple sequence alignments constructed using Cobalt [31], ClustalW [32], and T-Coffee [33] for HIV-1 IN (GB:AEA11266.1), PFV IN (PDB: 3L2Q:A), Ty3 IN (GB:AAA98435.1) (Fig. 1 and Table S1), and Moloney murine leukemia virus IN (GB: NP_955592.1). A PSI-BLAST search with these sequences plus ASV IN (PDB: 1CXQ:A) identified a gypsy retrotransposon IN-like sequence in the human genome, NP-060146.2, intermediate between PFV and Ty3 IN. The PFV IN CCD and other candidate domain boundaries were mapped onto NP-060146.2 and then onto the Ty3 IN primary sequence. These candidate boundaries were further analyzed using other bioinformatics tools, including Phyre [34], SAM-T08 [35], SMART [36], Jpred [37], PSIPRED [38], and Meta-TASSER [39]. The final domain boundary assignments represented a consensus based on these different approaches.

### 2. Directed Shuffling based Chimera Construction

Broadly speaking, there are two general methods that use PCR and bipartite PCR primers to assemble genes for chimeric proteins: (1) Splicing by Overlap Extension (SOEing) [40]–[44], and (2) fusion PCR [45], which is adapted here (literature nomenclature is inconsistent, and some SOEing methods are labeled fusion PCR [46]). In both methods, the bipartite primers are essentially the same: an upstream half that matches the downstream end of the upstream DNA chimeric fragment, followed by a downstream half that matches the upstream end of the downstream DNA chimeric fragment. The methods differ in the number and use of the bipartite primers, and in the way the DNA chimeric fragments are assembled into the final chimeric gene product. In the first SOEing step, each DNA chimeric fragment is amplified separately using the sense-strand bipartite primer at its upstream end and the antisense-strand bipartite primer at its downstream end. The result is double-stranded DNA (ds-DNA) chimeric fragments with flanking ds-DNA regions that overlap the adjacent ends of the upstream and downstream adjacent chimeric ds-DNA fragments. In the second SOEing step, the overlapping chimeric ds-DNA fragments are all PCR amplified together with end PCR primers for the final chimeric gene product. Both 3′ ends of each chimeric ds-DNA fragment extend first into their adjacent overlapping fragments and ultimately through the 5′ end of the final chimeric gene product, which is amplified by the end PCR primers. In contrast, in the first step of the fusion PCR strategy, only the sense (or only the anti-sense) bipartite primers are added to the native or recoded source genes. Bipartite primers are extended as single-stranded DNA (ss-DNA) first into their adjacent overlapping fragments and ultimately through the 5′ end of the final chimeric gene product. In the second step of this strategy, end primers for the final chimeric gene product are added, which amplify the final chimeric gene product. Thus, SOEing is potentially more reliable because the bipartite primers extend in both directions, and so the reaction succeeds if either primer extension succeeds; while fusion PCR is potentially more efficient because only one bipartite primer is required for each chimeric junction instead of two in SOEing, and because potentially fewer separate PCR reactions need be done than are required by SOEing.

We introduced several technical improvements into the original fusion PCR method [45]. Several fusion PCR reactions could be performed in the same tube at the same time to produce multi-part chimeras in the same reaction. *Dpn*I was added to digest the original template when the NTD and CTD domains were from the same parental gene. Perhaps most importantly, DNA and potential RNA secondary structures [47] were removed from the DNA sequences during IN gene recoding. In the case of DNA structures this reduced potential for deleterious cross-hybridization, which is known to degrade mutagenesis efficiency and correctness [48]. The presence of excessive DNA secondary structure may require very long oligos to insure unique hybridization, which in turn may lead to problems with primer dimers, primer hairpins, partial primer hybridization to the wrong gene location, etc. Elimination of potential RNA structures reduces the possibility of unanticipated consequences of non-native RNA structures on subsequent gene expression. Reducing DNA/RNA secondary structure and its concomitant cross-hybridization hazard allowed us to achieve the improved construction efficiency of fusion PCR without sacrificing the reliability of SOEing.

Here we used the CODA method [49] to remove DNA/RNA secondary structure, but any gene design software that removed gene secondary structure could be used to equivalent effect. First, the IN aa sequences of HIV, PFV, and Ty3 were joined end-to-end to create a large virtual aa sequence consisting of the aa sequences of all three IN proteins, one after the other. This large virtual aa sequence was recoded by the CODA design software using synonymous codon substitutions into a virtual DNA sequence (Table S2) encoding an identical virtual aa sequence, but with reduced DNA/RNA secondary structure and a Codon Adaptation Index (CAI) [50] optimized for expression in *S. cerevisiae, E. coli,* and human cells. The result was that every DNA location in every IN gene was assigned a globally unique thermodynamic address with respect to cross-hybridization [48], and consequently the bipartite primers used in chimera construction could be targeted reliably to their desired DNA location. Thereafter the virtual DNA sequence was again divided into the three DNA sequences for the individual IN genes of HIV, PFV, and Ty3.

These three IN genes were synthesized as described [48] and detailed in Methods S1. Oligonucleotides were chemically synthesized by Integrated DNA Technologies, Inc., (San Diego, CA) and used to produce the source DNA for subsequent chimera construction as described below. These three IN genes were cloned into plasmids using the 5′ *Nde*I site and 3′ *Xho*I site carried by the 5′ and 3′ end primers.

**Figure 2:**
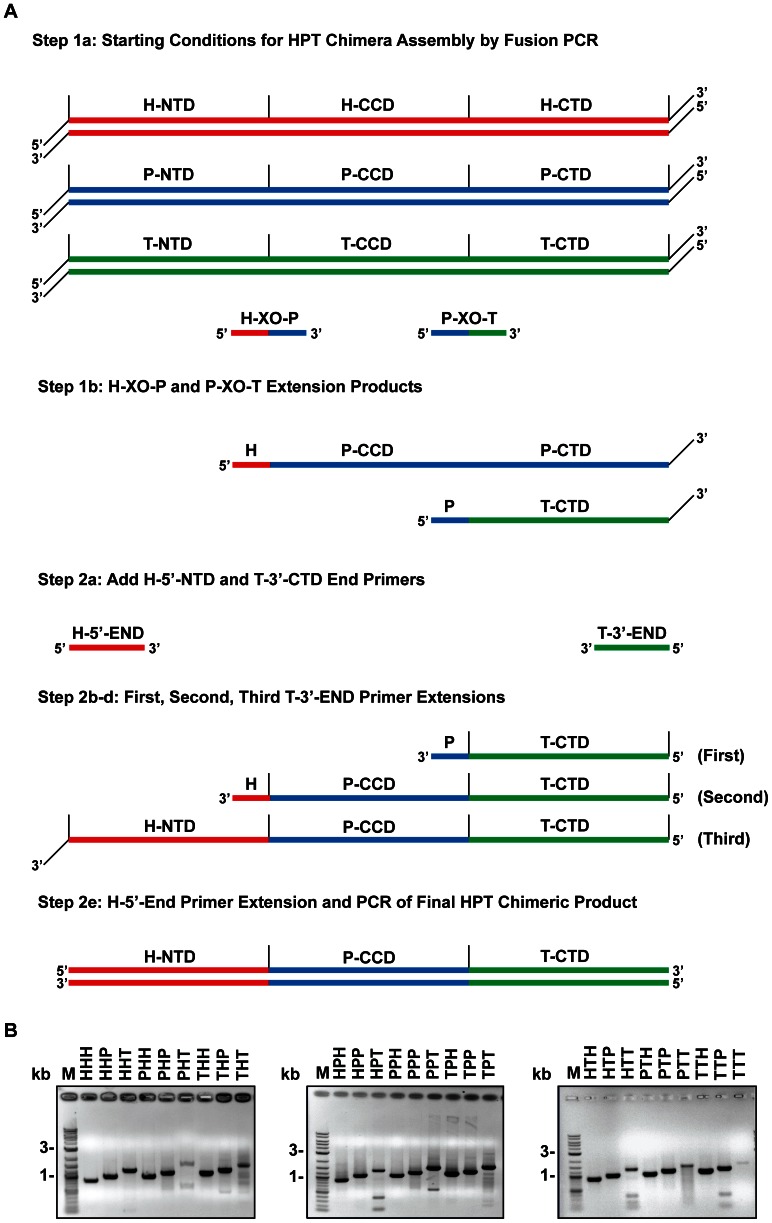
Production of CODA assembled chimeric IN coding sequences. **A. Example of crossover oligonucleotide-directed production of chimeric HTT IN-coding sequence.** Parental recoded HIV-1 and Ty3 IN cassettes in pCRII-Blunt-TOPO plasmids provided full-length, methylated “parental” DNA templates. Step 1, the bipartite Ty3-NTDxHIV1-CCD crossover oligonucleotide was extended by polymerization on the Ty3 template to produce a 5′-truncated coding strand. The complement (noncoding strand) of this DNA was produced by DNA polymerase extension of primer 1 annealed to the Ty3 downstream end. This truncated noncoding strand annealed to the HIV-1 template and a polymerase extension reaction yielded the full-length HTT noncoding strand. Step 2, primer 2 annealed to the noncoding HTT template and was extended to yield the full-length HTT coding strand. Terminal primers were present at 0.2 µM and the crossover primer at 0.04 µM. The full-length HTT chimeric product was amplified with the upstream HIV-1 primer 1 and downstream Ty3 primer 2. In cases where the NTD and CTD domains were from the same gene, *Dpn*I was added to the second reaction to remove the methylated parental DNA. B. Chimeric IN gene products. Final extension products from the assembly described in A were isolated by electrophoresis on 1% agarose gels. Chimeric sequences are identified using three letter codes: H, HIV-1; P, PFV; and T, Ty3; in the NTD-, CCD-, and CTD-coding regions respectively.

Directed shuffling [41] of the three IN domains by bipartite oligonucleotides (Fig. 2A) (Table S2) was used to produce the 24 possible chimeric IN genes. Two bipartite primers were designed for each pair of IN genes (one per chimera)(Ty3:HIV-1, HIV-1:PFV, and Ty3:PFV) at each domain boundary (NTD:CCD and CCD:CTD) (Table S4). The 5′ and 3′ end primers included *Nde*I and *Xho*I restriction sites, respectively (Supplemental Materials). Chimeras were constructed by 24 reactions, each of which involved two steps of polymerase extension (see Fig. 2A).

The first reaction step used the constituent IN plasmid gene templates and bipartite primers corresponding to the desired NTD, CCD, and CTD domains to be joined. The necessary template IN pCRII-Blunt-TOPO clones containing full-length IN (50 ng each) were added to a primer extension reaction composed of the appropriate bipartite primers at a final concentration of 0.2µM each, along with 2.5 U of *PfuUltra™* II Fusion HS DNA polymerase (Stratagene), 300 µM dNTPs (Fermentas), and 1X PfuUltra reaction buffer. These primer extension and PCR amplification reactions were performed in a thermal cycler using the following protocol: 10 min denaturation step at 95°C, followed by 30 cycles of 20 sec at 95°C, 20 sec at 62°C, and 40 sec at 72°C, and a final step of 5 min at 72°C.

The second reaction step used NTD and CTD end primers to amplify the chimeric product of the first reaction step. For chimeric protein whose NTD and CTD domains were from the same virus IN, 10 units of *Dpn*I was added after the first reaction step and incubated at 37°C for 2 hrs to eliminate the template. Next, 0.2µM of 5′ and 3′ end primers, 300 µM dNTPs (Fermentas, Waltham, MA), and an extra 2.5 U of *PfuUltra™* II Fusion HS DNA polymerase (Stratagene Corp., La Jolla, CA) were added. Another PCR reaction was performed to amplify the final chimera construct. These primer extension and PCR amplification reactions were performed in a thermal cycler using the same protocol as above.

The 5′ and 3′ end primers contained 5′ *Nde*I site and 3′ *Xho*I site. The full-length PCR products were purified with Qiagen PCR Purification Kit, digested with *Nde*I and *Xho*I, and ligated into *Nde*I and *Xho*I sites of pET29a. Full-length sequence was verified by DNA sequencing (Genewiz Inc., South Plainfield, NJ).

Each chimeric gene crossover was constructed by two polymerase extension reactions and products were amplified by PCR (Fig. 2A). These primer extension and PCR amplification reactions were performed in a thermal cycler using the following protocol: 10 min denaturation step at 95°C, followed by 30 cycles of 20 s at 95°C, 20 s at 59°C, and 40 s at 72°C, and a final step of 5 min at 72°C. PCR products were subjected to electrophoresis in 1% agarose gel (Fig. 2B). DNA products were purified with Qiagen PCR Purification Kit according to manufacturer’s instructions.

Although the 24 crossover products described here were generated in separate reactions for protein expression and assays, multi-crossover products also could be generated in a single reaction using two or more crossover oligonucleotides and multiple DNA templates for generating more complex libraries.

### 3. Protein Expression and Purification

The three native and twenty-four chimeric IN genes optimized for expression in *S. cerevisiae, E. coli* and humans were cloned into the *Nde*I and *Xho*I sites of pET29a (EMD Biosciences, San. Diego, CA). Proteins were expressed by isopropyl β-D-1-thiogalactopyranoside induction and purified as previously described [35], with modifications. Cell lysate in lysis buffer (20 mM HEPES pH 7.5, 1 mM EDTA, 5 mM beta-mercaptoethanol, 1 mM PMSF) was centrifuged at 20,000×g for 30 min at 4°C, and pellet was resuspended in solubilization buffer (20 mM HEPES pH7.5, 1 M NaCl, 10 mM CHAPS, 10% glycerol, 5 mM BME) for 1 h at 4°C to solubilize IN. After another centrifugation at 20,000×g for 30 min at 4°C, supernatant containing IN was further purified by nickel affinity chromatography [29].

### 4. In vitro Strand-transfer Assays

**Figure 3:**
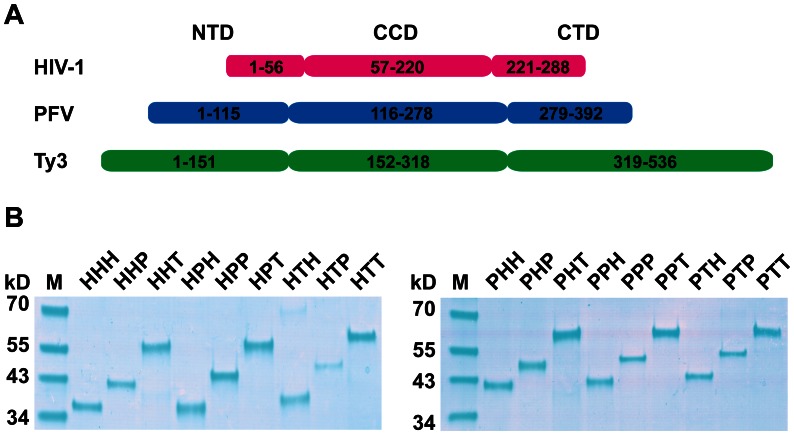
Expression of chimeric IN proteins. **A.** Composition of chimeric subdomains. **B.** Protein expression. Proteins were expressed in *E. coli*, purified by nickel affinity chromatography, fractionated on SDS 4–20% gradient polyacrylamide gels under denaturing conditions, and stained with Coomassie brilliant blue.

IN strand-transfer reactions were performed as described previously [29]. Samples included 50 fmole of target plasmid (pLY1855) containing the RNA Pol III-transcribed *SNR6* gene; 250 fmole of duplex DNA oligonucleotides containing one strand with a 5′ end complementary to one PCR primer and 3′ end representing HIV-1, PFV, or Ty3 cDNA terminal sequence (“donor substrate”); and 1000 fmole of HIV-1, PFV, Ty3 IN in a total volume of 40 µL (Table S3). Reactions were performed in the presence of Mn^2+^ or Mg^2+^. Some reactions were performed in the presence of 250 fmole of a synthetic Ty3 targeting protein, a fusion of Pol III transcription factors Brf1 and the TATA-binding protein (Brf-TBP-Brf), referred to as “triple fusion protein” (TFP) [29], [51]. Substrates consisted of 23 nts of a common 5′ end and an HIV-1 (19 nts), PFV (19 nts), or Ty3 (20 nts) specific U5 LTR 3′ end (Table S4). PCR to detect products used primers complementary to the substrate and to target plasmid pLY1855. PCR to normalize plasmid levels per reaction used primers complementary to the gene for β-lactamase carried on pLY1855 (Table S5) [52]. Plasmids containing Ty3 sequence upstream of *SNR6* on pXQ3659/pXQ3660 and pXQ3661 were used as controls for strand-transfer positions under MgCl_2_- and MnCl_2_- containing conditions, respectively [29] (Fig. 3A).

## Results

### 1. Prediction of Domain Boundaries of Ty3

Protein modeling was consistent with overall similarity of Ty3 and retroviral IN secondary structure within the CCD. Alignment of Ty3 IN allowed identification of CCD boundaries consistent with those established for HIV-1 and PFV IN [13]. However, alpha helices occur at the carboxyl-terminal ends of the CCD in HIV-1 and PFV IN and alpha helical structure was predicted in this region for Ty3 IN. Boundaries were therefore adjusted to retain the determined or predicted helices in the CCD domains of each protein (Fig. 1, Supplemental Material). This analysis positioned the Ty3 IN CCD from aa 152 to 318.

Preliminary experiments indicated poor expression correlated with the presence of a predicted RNA structure in the 5′ end of the PFV RNA and deletion of the first three codons of the PFV IN-coding sequence improved bacterial expression (data not shown). The final structural alignment upon which chimeras were designed compares HIV-1 IN aa 57–220/288, PFV IN aa 116–278/392, and Ty3 IN aa 152–318/536 (Fig. 1).

### 2. Computationally Optimized DNA Assembly of wt and Chimeric IN Genes

Synthetic HIV-1, PFV, and Ty3 IN genes were assembled and cloned into the expression vector, thereby fusing a vector His(6) coding sequence to the downstream end as described in Methods S1. Using these DNA templates in the two-step crossover PCR (Fig. 2A), 24 chimeric sequences were generated. Wt and chimeric products are referred to by domain source: H (HIV-1), P (PFV), or T (Ty3) in order of NTD, CCD, and CTD. Coding regions were confirmed by DNA sequencing. In all reactions, the major product was of the expected sequence. In only one reaction (PHT) was there a significant amount of off-target product (Fig. 2B). Although all chimeric proteins were expressed, the eight chimeras containing the Ty3 NTD were insoluble under all native conditions tested (Fig. 3A) (data not shown). Other proteins were purified using affinity chromatography as previously described for Ty3 IN (Fig. 3B) [29].

### 3. Strand-transfer Activity of Chimeric IN Proteins

**Figure 4:**
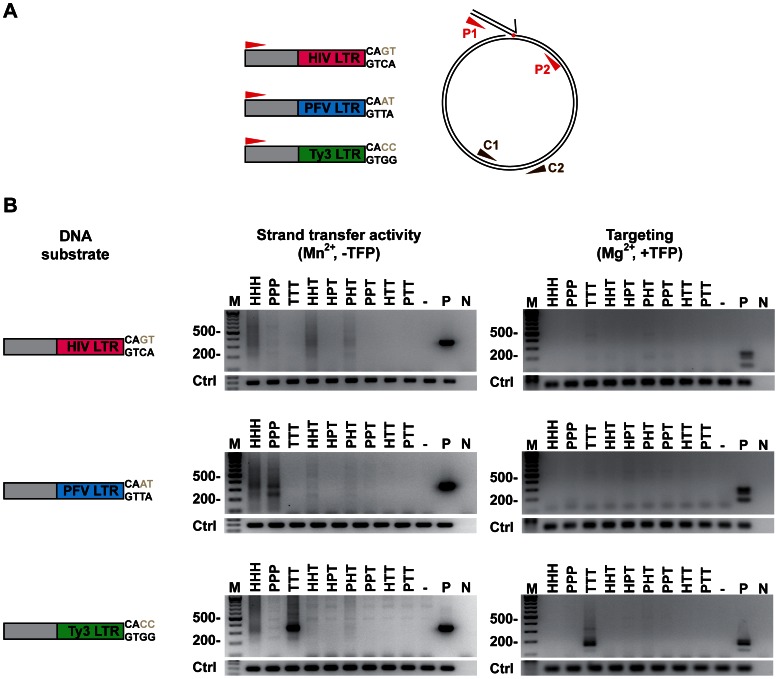
Strand-transfer assay of chimeric IN proteins. **A. Strand-transfer assay strategy.** HIV-1, PFV, and Ty3 IN proteins and chimeras were assayed and products were detected by PCR using primers annealed to the donor substrate (P1) and the target plasmid pLY1855 (P2) to control DNA per reaction (C1,C2), as described in Materials and Methods. **B. PCR products of strand-transfer assays.** Reactions were done under conditions where native Ty3 IN targets the strand transfer in a sequence specific mode (Mn^2+^, left panel) or in a position-specific mode (Mg^2+^ plus host factor TFP, right panel). Donor substrates represent preprocessed U5 ends of the HIV-1-, PFV-, and Ty3-cDNAs. Assay products were extracted and fractionated as described in Materials and Methods. The assays were performed a minimum of two times and representative products are shown. P, positive control PCR of Ty3 integrated in target plasmid; N, negative control *in vitro* reaction with no IN processed for PCR. Abbreviations are the same as in the Fig. 2 legend.

Based on observations with other targeted retrotransposons and *in vitro* pull-downs with Ty3 IN and targeting protein TFP (manuscript in preparation), the Ty3 IN CTD was a candidate for mediating target protein interactions. Therefore the wt (HHH, PPP, and TTT) and six chimeric IN proteins containing Ty3 IN CTD (HHT, HPT, PHT, PPT, HTT, and PTT) were assayed in the presence of Mn^2+^ or Mg^2+^with homologous and heterologous donor substrates to test whether the Ty3 CTD was sufficient to confer targeting specificity. *In vitro* strand-transfer activity of chimeric IN proteins was monitored as the transfer of preprocessed duplex (3′ end recessed) oligonucleotide substrate into a target plasmid and detection of the product by PCR such that the size of the amplicon reflected the position of strand transfer (Fig. 4A).

Mn^2+^ enhances some retrovirus IN activities [53], but in the case of Ty3 IN it caused strand transfer to favor regions with sequence similar to the 8-bp perfect inverted repeat of the native donor Ty3 cDNA [29] (Fig. 4B, left panel). Native HIV-1 IN generated products with all three substrates in Mn^2+^. Native PFV and Ty3 IN displayed activity only with homologous donor substrates. As previously reported, Ty3 IN-generated strand transfer products concentrated near a sequence in the plasmid which resembles the Ty3 cDNA termini [29]. To a lesser extent the assays of PFV and HIV-1 IN proteins showed some clustered strand transfer. However, these PFV and HIV-1 PCR products were not further investigated and therefore could also represent bias in the PCR amplification reaction. Notably, chimeras with HIV-1 CCD showed detectable activity with all three substrates.

Reactions were also conducted in Mg^2+^, which allows Ty3 position-specific integration in the presence of TFP [29] (Fig. 4B, right panel). In the presence of Mg^2+^ and the TFP targeting factor, only native Ty3 IN showed position specific activity. The major site of strand transfer for Ty3 IN was the site of *SNR6* transcription initiation mediated by TFP, as previously verified by sequencing. No chimeric IN showed position-specific strand transfer.

## Discussion

Application of CODA was previously demonstrated for rapid *in vitro* assembly of recoded gene sequences [49] and for scanning saturation mutagenesis [54]. In this work we demonstrated a new application of CODA sequences in generation of specific user-directed crossover libraries.

Crossover points were chosen based on PFV and HIV-1 IN structures and modeling of the Ty3 IN CCD to identify domain boundaries compatible with the retrovirus IN proteins [13]. In Mn^2+^ each native recoded CODA enzyme was active on homologous donor substrates, but only HIV-1 IN was active on heterologous substrates. This result is consistent with previous work showing that HIV-1 IN is not highly specific with respect to donor substrate DNA, particularly in the presence of Mn^2+^ [55]. Although the specific activity of these proteins was not determined, HIV-1 IN generated more detectable products on the PFV substrate than on the Ty3 substrate. Comparison of the terminal plus strand ten nts of U5 DNA sequence, which includes positions implicated in IN processing specificity (HIV-1:AAAATCTCTAGCA; PFV:AAAATTCCATACA; and Ty3: GCCCGTAATACAACA) [2], [55], shows that Ty3 differs dramatically from HIV-1 and PFV in these terminal sequences. These results also indicate that if HIV-1, similar to PFV, contacts target DNA with CCD and CTD domains, then the HIV-1 CCD and Ty3 CTD are compatible with respect to requisite contacts. The lack of activity of chimeras with PFV or Ty3 CCD might result from steric clashes between the domains, requirement for the NTD in addition to one other domain, or requirement for all three domains to cooperate in generating the position specific product.

Previous analyses of chimeras between HIV-1 and other lenti-retroviruses, including feline immunodeficiency virus [56], [57], Visna [58], [59], caprine arthritis encephalitis virus [58], and PFV [60] have used similar divisions of IN into NTD, CCD, and CTD domains to map viral substrate and local target DNA specificity. These studies, consistent with more recent insights from PFV structures, largely agree that retroviral cDNA substrate specificity is not affected by the NTD and is determined by the CCD with contributions from the CTD. Chimeras of HIV-1 and visna IN proteins showed parental patterns of target interaction associated with the CCD in alcoholysis assays but did not recreate parental IN patterns with cDNA substrates [59], [61]. Chimeras between HIV-1 and feline immunodeficiency virus showed strong influence of the CCD on strand transfer patterns [57] and so are consistent with PFV structures showing contacts between the CCD and target DNA [12], [18]–[20]. HIV and PFV chimeras, with slightly different CCD bounds than used in our study, were more informative with respect to cDNA end recognition than local sequence targeting because of weak strand transfer activity [60]. The current study attempted to map interactions responsible for docking IN on the target cDNA as well as interactions responsible for Ty3 local target-DNA specificity in Mn^2+^.

In our assays in the presence of Mg^2+^ and targeting protein TFP, only Ty3 IN generated a product. Lack of chimeric IN products was most meaningful in the case of the chimeras containing the HHT and PHT, which were active in the presence of Mn^2+^ indicating that they were grossly competent for strand transfer. Because HIV CCD alone is competent for disintegration, but not strand transfer, this result is consistent with some contribution of the Ty3 and PFV domains to the strand transfer activity observed for the HHT and PHT chimeras. Although *in vitro* pull-down assays show that the Ty3 CCD and CTD interact independently with the targeting factor TFP (manuscript in preparation), HTT and PTT chimeras also failed to show strand transfer activity. This was expected based on the lack of TFP-independent activity in Mn^2+^-containing assays.

In summary, this work demonstrated a DNA-directed crossover method for generation of chimeric proteins, useful in structure-function studies and in the development of novel combinations of protein domains. Assays of chimeric IN proteins representing two lentiviruses and the Ty3 retrotransposon demonstrated that Ty3 IN strand transfer, unlike that of HIV-1, is restricted for cDNA terminal sequences; that HIV-1 NTD and CCD are compatible with the Ty3 CTD for utilization of the HIV-1 substrate; and that PFV may be similar to Ty3 in exhibiting strong sequence based targeting in the presence of Mn^2+^.

## Supporting Information

Methods S1 (DOCX)

Table S1 Amino acid sequences of wild-type integrases.
(DOCX)

Table S2 CODA DNA sequences of wild type integrases.
(DOCX)

Table S3 Oligonucleotides for integrase gene assembly.
(DOCX)

Table S4 Oligonucleotides used in crossover PCR.
(DOCX)

Table S5 Oligonucleotides used for *in vitro* strand-transfer assay.
(DOCX)

## References

[1] Chalker DL, Sandmeyer SB (1992) Ty3 integrates within the region of RNA polymerase III transcription initiation. Genes Dev 6: 117–128. 1309715 10.1101/gad.6.1.117

[2] Li X, Krishnan L, Cherepanov P, Engelman A (2011) Structural biology of retroviral DNA integration. Virology 411: 194–205. 21216426 10.1016/j.virol.2010.12.008 PMC3640404

[3] Bushman F, Lewinski M, Ciuffi A, Barr S, Leipzig J, et al (2005) Genome-wide analysis of retroviral DNA integration. Nat Rev Microbiol 3: 848–858. 16175173 10.1038/nrmicro1263

[4] Engelman A, Mizuuchi K, Craigie R (1991) HIV-1 DNA integration: mechanism of viral DNA cleavage and DNA strand transfer. Cell 67: 1211–1221. 1760846 10.1016/0092-8674(91)90297-c

[5] Craigie R (2001) HIV integrase, a brief overview from chemistry to therapeutics. J Biol Chem 276: 23213–23216. 11346660 10.1074/jbc.R100027200

[6] Engelman A, Craigie R (1992) Identification of conserved amino acid residues critical for human immunodeficiency virus type 1 integrase function in vitro. J Virol 66: 6361–6369. 1404595 10.1128/jvi.66.11.6361-6369.1992 PMC240128

[7] Skalka AM (1993) Retroviral DNA integration: lessons for transposon shuffling. Gene 135: 175–182. 8276256 10.1016/0378-1119(93)90063-9

[8] Chow SA, Vincent KA, Ellison V, Brown PO (1992) Reversal of integration and DNA splicing mediated by integrase of human immunodeficiency virus. Science 255: 723–726. 1738845 10.1126/science.1738845

[9] Bushman FD, Engelman A, Palmer I, Wingfield P, Craigie R (1993) Domains of the integrase protein of human immunodeficiency virus type 1 responsible for polynucleotidyl transfer and zinc binding. Proc Natl Acad Sci U S A 90: 3428–3432. 8386373 10.1073/pnas.90.8.3428 PMC46313

[10] Li M, Mizuuchi M, Burke TR Jr, Craigie R (2006) Retroviral DNA integration: reaction pathway and critical intermediates. EMBO J 25: 1295–1304. 16482214 10.1038/sj.emboj.7601005 PMC1422164

[11] Valkov E, Gupta SS, Hare S, Helander A, Roversi P, et al (2009) Functional and structural characterization of the integrase from the prototype foamy virus. Nucleic Acids Res 37: 243–255. 19036793 10.1093/nar/gkn938 PMC2615609

[12] Maertens GN, Hare S, Cherepanov P (2010) The mechanism of retroviral integration from X-ray structures of its key intermediates. Nature 468: 326–329. 21068843 10.1038/nature09517 PMC2999894

[13] Hare S, Gupta SS, Valkov E, Engelman A, Cherepanov P (2010) Retroviral intasome assembly and inhibition of DNA strand transfer. Nature 464: 232–236. 20118915 10.1038/nature08784 PMC2837123

[14] Dyda F, Hickman AB, Jenkins TM, Engelman A, Craigie R, et al (1994) Crystal structure of the catalytic domain of HIV-1 integrase: similarity to other polynucleotidyl transferases. Science 266: 1981–1986. 7801124 10.1126/science.7801124

[15] Krishnan L, Li X, Naraharisetty HL, Hare S, Cherepanov P, et al (2010) Structure-based modeling of the functional HIV-1 intasome and its inhibition. Proc Natl Acad Sci U S A 107: 15910–15915. 20733078 10.1073/pnas.1002346107 PMC2936642

[16] Lewinski MK, Bisgrove D, Shinn P, Chen H, Hoffmann C, et al (2005) Genome-wide analysis of chromosomal features repressing human immunodeficiency virus transcription. J Virol 79: 6610–6619. 15890899 10.1128/JVI.79.11.6610-6619.2005 PMC1112149

[17] Maertens G, Cherepanov P, Pluymers W, Busschots K, De Clercq E, et al (2003) LEDGF/p75 is essential for nuclear and chromosomal targeting of HIV-1 integrase in human cells. J Biol Chem 278: 33528–33539. 12796494 10.1074/jbc.M303594200

[18] Cherepanov P, Ambrosio AL, Rahman S, Ellenberger T, Engelman A (2005) Structural basis for the recognition between HIV-1 integrase and transcriptional coactivator p75. Proc Natl Acad Sci U S A 102: 17308–17313. 16260736 10.1073/pnas.0506924102 PMC1297672

[19] Vanegas M, Llano M, Delgado S, Thompson D, Peretz M, et al (2005) Identification of the LEDGF/p75 HIV-1 integrase-interaction domain and NLS reveals NLS-independent chromatin tethering. J Cell Sci 118: 1733–1743. 15797927 10.1242/jcs.02299

[20] Busschots K, Vercammen J, Emiliani S, Benarous R, Engelborghs Y, et al (2005) The interaction of LEDGF/p75 with integrase is lentivirus-specific and promotes DNA binding. J Biol Chem 280: 17841–17847. 15749713 10.1074/jbc.M411681200

[21] Levin HL, Moran JV (2011) Dynamic interactions between transposable elements and their hosts. Nat Rev Genet 12: 615–627. 21850042 10.1038/nrg3030 PMC3192332

[22] Devine SE, Boeke JD (1996) Integration of the yeast retrotransposon Ty1 is targeted to regions upstream of genes transcribed by RNA polymerase III. Genes Dev 10: 620–633. 8598291 10.1101/gad.10.5.620

[23] Xie W, Gai X, Zhu Y, Zappulla DC, Sternglanz R, et al (2001) Targeting of the yeast Ty5 retrotransposon to silent chromatin is mediated by interactions between integrase and Sir4p. Mol Cell Biol 21: 6606–6614. 11533248 10.1128/MCB.21.19.6606-6614.2001 PMC99806

[24] Malik HS, Eickbush TH (1999) Modular evolution of the integrase domain in the Ty3/Gypsy class of LTR retrotransposons. J Virol 73: 5186–5190. 10233986 10.1128/jvi.73.6.5186-5190.1999 PMC112568

[25] Gao X, Hou Y, Ebina H, Levin HL, Voytas DF (2008) Chromodomains direct integration of retrotransposons to heterochromatin. Genome Res 18: 359–369. 18256242 10.1101/gr.7146408 PMC2259100

[26] Kordis D (2005) A genomic perspective on the chromodomain-containing retrotransposons: Chromoviruses. Gene 347: 161–173. 15777633 10.1016/j.gene.2004.12.017

[27] Singleton TL, Levin HL (2002) A long terminal repeat retrotransposon of fission yeast has strong preferences for specific sites of insertion. Eukaryot Cell 1: 44–55. 12455970 10.1128/EC.01.1.44-55.2002 PMC118054

[28] Behrens R, Hayles J, Nurse P (2000) Fission yeast retrotransposon Tf1 integration is targeted to 5′ ends of open reading frames. Nucleic Acids Res 28: 4709–4716. 11095681 10.1093/nar/28.23.4709 PMC115174

[29] Qi X, Sandmeyer SB (2012) In vitro targeting of strand transfer by the Ty3 retroelement integrase. J Biol Chem 287: 18589–18595. 22493285 10.1074/jbc.M111.326025 PMC3365781

[30] Engelman A, Bushman FD, Craigie R (1993) Identification of discrete functional domains of HIV-1 integrase and their organization within an active multimeric complex. EMBO J 12: 3269–3275. 8344264 10.1002/j.1460-2075.1993.tb05996.x PMC413594

[31] Papadopoulos JS, Agarwala R (2007) COBALT: constraint-based alignment tool for multiple protein sequences. Bioinformatics 23: 1073–1079. 17332019 10.1093/bioinformatics/btm076

[32] Thompson JD, Higgins DG, Gibson TJ (1994) CLUSTAL W: improving the sensitivity of progressive multiple sequence alignment through sequence weighting, position-specific gap penalties and weight matrix choice. Nucleic Acids Res 22: 4673–4680. 7984417 10.1093/nar/22.22.4673 PMC308517

[33] Notredame C, Higgins DG, Heringa J (2000) T-Coffee: A novel method for fast and accurate multiple sequence alignment. J Mol Biol 302: 205–217. 10964570 10.1006/jmbi.2000.4042

[34] Kelley LA, Sternberg MJ (2009) Protein structure prediction on the Web: a case study using the Phyre server. Nat Protoc 4: 363–371. 19247286 10.1038/nprot.2009.2

[35] Karplus K (2009) SAM-T08, HMM-based protein structure prediction. Nucleic Acids Res 37: W492–497. 19483096 10.1093/nar/gkp403 PMC2703928

[36] Ponting CP, Schultz J, Milpetz F, Bork P (1999) SMART: identification and annotation of domains from signalling and extracellular protein sequences. Nucleic Acids Res 27: 229–232. 9847187 10.1093/nar/27.1.229 PMC148142

[37] Cole C, Barber JD, Barton GJ (2008) The Jpred 3 secondary structure prediction server. Nucleic Acids Res 36: W197–201. 18463136 10.1093/nar/gkn238 PMC2447793

[38] McGuffin LJ, Bryson K, Jones DT (2000) The PSIPRED protein structure prediction server. Bioinformatics 16: 404–405. 10869041 10.1093/bioinformatics/16.4.404

[39] Zhou H, Pandit SB, Skolnick J (2009) Performance of the Pro-sp3-TASSER server in CASP8. Proteins 77 Suppl 9123–127. 19639638 10.1002/prot.22501 PMC2785221

[40] Ho SN, Hunt HD, Horton RM, Pullen JK, Pease LR (1989) Site-directed mutagenesis by overlap extension using the polymerase chain reaction. Gene 77: 51–59. 2744487 10.1016/0378-1119(89)90358-2

[41] Horton RM, Hunt HD, Ho SN, Pullen JK, Pease LR (1989) Engineering hybrid genes without the use of restriction enzymes: gene splicing by overlap extension. Gene 77: 61–68. 2744488 10.1016/0378-1119(89)90359-4

[42] Aamodt K, Abelev B, Abrahantes Quintana A, Adamova D, Adare AM, et al (2012) Particle-yield modification in jetlike azimuthal dihadron correlations in Pb-Pb collisions at radicals(NN) = 2.76 TeV. Phys Rev Lett 108: 092301. 22463626 10.1103/PhysRevLett.108.092301

[43] Quan J, Tian J (2009) Circular polymerase extension cloning of complex gene libraries and pathways. PLoS One 4: e6441. 19649325 10.1371/journal.pone.0006441 PMC2713398

[44] Quan J, Tian J (2011) Circular polymerase extension cloning for high-throughput cloning of complex and combinatorial DNA libraries. Nat Protoc 6: 242–251. 21293463 10.1038/nprot.2010.181

[45] Yon J, Fried M (1989) Precise gene fusion by PCR. Nucleic Acids Res 17: 4895. 2748349 10.1093/nar/17.12.4895 PMC318059

[46] Morita K, Tadano M, Nakaji S, Kosai K, Mathenge EG, et al (2001) Locus of a virus neutralization epitope on the Japanese encephalitis virus envelope protein determined by use of long PCR-based region-specific random mutagenesis. Virology 287: 417–426. 11531418 10.1006/viro.2001.1048

[47] Zuker M (2003) Mfold web server for nucleic acid folding and hybridization prediction. Nucleic Acids Res 31: 3406–3415. 12824337 10.1093/nar/gkg595 PMC169194

[48] Wassman CD, Tam PY, Lathrop RH, Weiss GA (2004) Predicting oligonucleotide-directed mutagenesis failures in protein engineering. Nucleic Acids Res 32: 6407–6413. 15585664 10.1093/nar/gkh977 PMC535685

[49] Larsen LS, Wassman CD, Hatfield GW, Lathrop RH (2008) Computationally Optimised DNA Assembly of synthetic genes. Int J Bioinform Res Appl 4: 324–336. 18640907 10.1504/IJBRA.2008.019578 PMC2668710

[50] Sharp PM, Li WH (1987) The rate of synonymous substitution in enterobacterial genes is inversely related to codon usage bias. Mol Biol Evol 4: 222–230. 3328816 10.1093/oxfordjournals.molbev.a040443

[51] Kassavetis GA, Soragni E, Driscoll R, Geiduschek EP (2005) Reconfiguring the connectivity of a multiprotein complex: fusions of yeast TATA-binding protein with Brf1, and the function of transcription factor IIIB. Proc Natl Acad Sci U S A 102: 15406–15411. 16227432 10.1073/pnas.0507653102 PMC1266137

[52] Yieh L, Kassavetis G, Geiduschek EP, Sandmeyer SB (2000) The Brf and TATA-binding protein subunits of the RNA polymerase III transcription factor IIIB mediate position-specific integration of the gypsy-like element, Ty3. J Biol Chem 275: 29800–29807. 10882723 10.1074/jbc.M003149200

[53] Hickman AB, Palmer I, Engelman A, Craigie R, Wingfield P (1994) Biophysical and enzymatic properties of the catalytic domain of HIV-1 integrase. J Biol Chem 269: 29279–29287. 7961898

[54] Baronio R, Danziger SA, Hall LV, Salmon K, Hatfield GW, et al (2010) All-codon scanning identifies p53 cancer rescue mutations. Nucleic Acids Res 38: 7079–7088. 20581117 10.1093/nar/gkq571 PMC2978351

[55] Esposito D, Craigie R (1998) Sequence specificity of viral end DNA binding by HIV-1 integrase reveals critical regions for protein-DNA interaction. EMBO J 17: 5832–5843. 9755183 10.1093/emboj/17.19.5832 PMC1170911

[56] Shibagaki Y, Chow SA (1997) Central core domain of retroviral integrase is responsible for target site selection. J Biol Chem 272: 8361–8369. 9079660 10.1074/jbc.272.13.8361

[57] Appa RS, Shin CG, Lee P, Chow SA (2001) Role of the nonspecific DNA-binding region and alpha helices within the core domain of retroviral integrase in selecting target DNA sites for integration. J Biol Chem 276: 45848–45855. 11585830 10.1074/jbc.M107365200

[58] Berger N, Heller AE, Stormann KD, Pfaff E (2001) Characterization of chimeric enzymes between caprine arthritis–encephalitis virus, maedi–visna virus and human immunodeficiency virus type 1 integrases expressed in Escherichia coli. J Gen Virol 82: 139–148. 11125167 10.1099/0022-1317-82-1-139

[59] Katzman M, Sudol M, Pufnock JS, Zeto S, Skinner LM (2000) Mapping target site selection for the non-specific nuclease activities of retroviral integrase. Virus Res 66: 87–100. 10653920 10.1016/s0168-1702(99)00126-4

[60] Lee HS, Kang SY, Shin CG (2005) Characterization of the functional domains of human foamy virus integrase using chimeric integrases. Mol Cells 19: 246–255. 15879710

[61] Katzman M, Sudol M (1995) Mapping domains of retroviral integrase responsible for viral DNA specificity and target site selection by analysis of chimeras between human immunodeficiency virus type 1 and visna virus integrases. J Virol 69: 5687–5696. 7637015 10.1128/jvi.69.9.5687-5696.1995 PMC189427

