# Supplementary material for: Directed DNA Shuffling of Retrovirus and Retrotransposon Integrase Protein Domains
Source: PLoS One. 2013 May 17;8(5):e63957. doi: 10.1371/journal.pone.0063957 (PMC3656877; doi:10.1371/journal.pone.0063957)
Supplement: Methods S1 — (DOCX) [file pone.0063957.s001.docx]

**Methods S1**

Gene Assembly

The three IN genes were assembled as described by Larsen et al.[[1](#_ENREF_1)]. The assembly method is hierarchical. PCR is used in several steps to extend overlapping DNA fragments to ever-longer DNA fragments. The process begins with purchased single-stranded oligonucleotides of approximately 60 bases in length, which entirely cover both strands of the entire gene. Adjacent oligonucleotides on the same strand abutt each other directly with no gaps, while adjacent oligonucleotides on opposite strands overlap each other by approximately 30 bases. In the first step of the process, groups of approximately 10 contiguous oligonucleotides from both strands are extended by PCR to produce double-stranded DNA fragments of approximately 300-350 bases in length (called “primary fragments [PF]”). The oligonucleotide groupings are chosen so that adjacent primary fragments overlap by approximately 90 bp (two overlapping oligonucleotides on opposite strands). In the second step, three contiguous overlapping primary fragments are extended by PCR to produce double-stranded DNA fragments of approximately 800-850 bases in length (called “secondary fragments [SF]”). Adjacent secondary fragments overlap each other by one primary fragment, or approximately 300-350 bases. In the final step, the overlapping secondary fragments are extended by PCR to produce the final gene with flanking sequences that facilitate subsequent cloning.

Assembly of each primary DNA fragment was performed as shown in Table S3. There, every purchased single-stranded DNA oligonucleotide is listed (On1 to On122) , along with whether it is on the forward (fwd) or reverse (rev) strand; in which primary fragments it is used (Pf1 to Pf15; “Pfx-y” means that the oligonucleotide is used in primary fragment number “x” as oligonucleotide number “y”) ; and its sequence. Some oligonucleotides are used in two adjacent primary fragments, and implement their overlap (e.g., On9 fwd [Pf1-9 Pf2-1] and On10 rev [Pf1-10 Pf2-2] occur in both primary fragment 1 and primary fragment 2, and consequently those two primary fragments overlap across those two oligonucleotides).

Ty3 IN was made from secondary fragment numbers 1-3. Secondary fragment number 1 was made from primary fragment numbers 1-3. Secondary fragment number 2 was made from primary fragment numbers 3-5. Secondary fragment number 3 was made from primary fragment numbers 5-7.

HIV IN was made from secondary fragment number s 4 and 5. Secondary fragment number 4 was made from primary fragment numbers 7-9. Secondary fragment number 5 was made from primary fragment numbers 9-11.

PFV IN was made from secondary fragment number s 6 and 7. Secondary fragment number 6 was made from primary fragment numbers 11-13. Secondary fragment number 7 was made from primary fragment numbers 13-15.

In each case, the final gene with flanking sequences appropriate for cloning was isolated by a PCR reaction with appropriate 3’ and 5’ primers.

First Step: Assembly of Oligonucleotides into Primary DNA Fragments (PF)

In the first step, primary DNA fragments were constructed by polymerase extension of short overlapping and abutting oligonucleotides approximately 60 bases in length. Oligonucleotides on opposing strands were overlapped by approximately 30 bases. For each primary DNA fragment, the constituent oligonucleotide set was added to a primer extension reaction at a final concentration of 0.04 µM along with an excess (0.2 µM) of leader (5’) and trailer (3’) primer oligonucleotide (the most 5’- and 3’-distant oligonucleotides). Each oligonucleotide set was extended into a primary DNA fragment with 2.5 U of *PfuUltra™* II Fusion HS DNA polymerase (Stratagene), 300 µM dNTPs (Fermentas), and 1X PfuUltra reaction buffer. These primer extension and PCR amplification reactions were performed in a thermal cycler using the following protocol: 10 min denaturation step at 95 ºC, followed by 30 cycles of 20 s at 95 ºC, 20 s at 59 ºC, and 40 s at 72 ºC, and a final step of 5 min at 72 ºC. PCR products were subjected to electrophoresis in 1% agarose gel. DNA products were purified with Qiagen PCR Purification Kit (Qiagen) according to manufacturer’s instructions.

Second Step: Assembly of Secondary DNA Fragments (SF)

In the second step, secondary DNA fragments were constructed by polymerase extension of three overlapping contiguous primary fragments from the first step. For each secondary DNA fragment, the three constituent primary DNA fragments were added to a primer extension reaction at a final concentration of 2 nM along with an excess (0.2 µM) of leader (5’) and trailer (3’) oligonucleotides as primers. The constituent primary DNA fragments were extended with 2.5 U of *PfuUltra™* II Fusion HS DNA polymerase, 300µM dNTPs, and 1X PfuUltra reaction buffer. These primer extension and PCR amplification reactions were performed in a thermal cycler using the following protocol: 10 min denaturation step at 95 ºC, followed by 30 cycles of 20 s at 95 ºC, 20 s at 62ºC, and 40 s at 72 ºC, and a final step of 5 min at 72 ºC. PCR products were subjected to electrophoresis in 1% agarose gel. DNA products were purified with Qiagen PCR Purification Kit according to manufacturer’s instructions.

Construction of the Full-Length Gene

Full length DNA products for each full-length IN gene were constructed by polymerase extension of their constituent secondary DNA fragments. For each full IN gene, each of the constituent SF DNA PCR products were added to a primer extension reaction at a final concentration of 2 nM along with an excess (0.2 µM) of leader (5’) and trailer (3’) primers. Each full-length IN gene was isolated by primer extension and PCR amplification with 2.5 U of PfuUltra High-Fidelity DNA polymerase, 300 µM dNTPs, and 1X PfuUltra buffer. These primer extension and PCR amplification reactions were performed as described above for secondary DNA fragment assembly. The PCR products were subjected to electrophoresis in 1% agarose gel. Each full-length gene was cloned into a pCRII-Blunt-TOPO vector (Invitrogen) according to the manufacturer’s protocol. The full-length gene sequence was verified by DNA sequencing.

1. Larsen LS, Wassman CD, Hatfield GW, Lathrop RH (2008) Computationally Optimised DNA Assembly of synthetic genes. Int J Bioinform Res Appl 4: 324-336.
