# Supplementary material for: Directed DNA Shuffling of Retrovirus and Retrotransposon Integrase Protein Domains
Source: PLoS One. 2013 May 17;8(5):e63957. doi: 10.1371/journal.pone.0063957 (PMC3656877; doi:10.1371/journal.pone.0063957)
Supplement: Table S1 — Amino acid sequences of wild-type integrases. (DOCX) [file pone.0063957.s002.docx]

**Table S1. Amino acid sequences of wild-type integrases (CCD domains are highlighted).**

**>HHH**

FLDGIDKAQEEHEKYHSNWRAMASDFNLPPVVAKEIVASCDKCQLKGEAMHGQVDCSP

GIWQLDCTHLEGKVILVAVHVASGYIEAEVIPAETGQETAYFLLKLAGRWPVKTVHTDNG

SNFTSTTVKAACWWAGIKQEFGIPYNPQSQGVIESMNKELKKIIGQVRDQAEHLKTAVQM

AVFIHNFKRKGGIGGYSAGERIVDIIATDIQTKELQKQITKIQNFRVYYRDSRDPVWKGP

AKLLWKGEGAVVIQDNSDIKVVPRRKAKIIRDYGKQMAGDDCVASRQDED

**>PPP**

CNTKKPNLDAELDQLLQGHYIKGYPKQYTYFLEDGKVKVSRPEGVKIIPPQSDRQKIV

LQAHNLAHTGREATLLKIANLYWWPNMRKDVVKQLGRCQQCLITNASNKASGPILRPDRP

QKPFDKFFIDYIGPLPPSQGYLYVLVVVDGMTGFTWLYPTKAPSTSATVKSLNVLTSIAI

PKVIHSDQGAAFTSSTFAEWAKERGIHLEFSTPYHPQSSGKVERKNSDIKRLLTKLLVGR

PTKWYDLLPVVQLALNNTYSPVLKYTPHQLLFGIDSNTPFANQDTLDLTREEELSLLQEI

RTSLYHPSTPPASSRSWSPVVGQLVQERVARPASLRPRWHKPSTVLKVLNPRTVVILDHL

GNNRTVSIDNLKPTSHQNGTTNDTATMDHLEKNE

**>TTT**

TITPETSRPIDTESWKSYYKSDPLCSAVLIHMKELTQHNVTPEDMSAFRSYQKKLELS

ETFRKNYSLEDEMIYYQDRLVVPIKQQNAVMRLYHDHTLFGGHFGVTVTLAKISPIYYWP

KLQHSIIQYIRTCVQCQLIKSHRPRLHGLLQPLPIAEGRWLDISMDFVTGLPPTSNNLNM

ILVVVDRFSKRAHFIATRKTLDATQLIDLLFRYIFSYHGFPRTITSDRDVRMTADKYQEL

TKRLGIKSTMSSANHPQTDGQSERTIQTLNRLLRAYASTNIQNWHVYLPQIEFVYNSTPT

RTLGKSPFEIDLGYLPNTPAIKSDDEVNARSFTAVELAKHLKALTIQTKEQLEHAQIEME

TNNNQRRKPLLLNIGDHVLVHRDAYFKKGAYMKVQQIYVGPFRVVKKINDNAYELDLNSH

KKKHRVINVQFLKKFVYRPDAYPKNKPISSTERIKRAHEVTALIGIDTTHKTYLCHMQDV

DPTLSVEYSEAEFCQIPERTRRSILANFRQLYETQDNPEREEDVVSQNEICQYDNTSP
