# Supplementary material for: Directed DNA Shuffling of Retrovirus and Retrotransposon Integrase Protein Domains
Source: PLoS One. 2013 May 17;8(5):e63957. doi: 10.1371/journal.pone.0063957 (PMC3656877; doi:10.1371/journal.pone.0063957)
Supplement: Table S2 — CODA DNA sequences of wild type integrases. (DOCX) [file pone.0063957.s003.docx]

**Table S2. CODA DNA sequences of wild type integrases (CCD domains are highlighted).**

**>HHH**

TTCCTGGATGGCATCGATAAAGCTCAGGAAGAACACGAAAAATACCACTCTAACTGGCGTGCAATGGCTTCTGATTTCAACTTGCCACCTGTTGTTGCAAAAGAAATCGTTGCTTCCTGCGACAAATGTCAACTGAAGGGTGAAGCTATGCACGGTCAGGTTGATTGCTCTCCAGGTATTTGGCAGTTGGATTGCACTCACCTGGAAGGGAAAGTAATTCTGGTTGCTGTTCACGTAGCTTCTGGTTACATTGAAGCTGAGGTTATTCCGGCTGAAACTGGTCAGGAAACTGCTTATTTCTTGCTGAAATTGGCAGGTCGCTGGCCGGTTAAAACTGTTCACACTGATAACGGTTCCAACTTCACTTCTACTACTGTTAAAGCTGCATGTTGGTGGGCTGGTATCAAACAGGAATTTGGTATTCCATACAACCCACAGTCCCAAGGCGTTATTGAATCTATGAACAAAGAGCTGAAAAAGATTATTGGTCAAGTTCGTGATCAGGCTGAACACCTGAAAACTGCTGTGCAGATGGCAGTATTCATTCACAACTTCAAACGTAAAGGTGGTATTGGTGGTTACAGCGCAGGTGAACGCATTGTTGATATTATTGCAACTGACATTCAAACCAAGGAATTGCAGAAACAGATTACCAAGATTCAGAACTTCCGTGTTTACTACCGTGATTCTCGTGATCCAGTTTGGAAAGGCCCAGCTAAACTGTTGTGGAAAGGTGAAGGTGCTGTTGTTATCCAGGATAACTCTGATATTAAAGTTGTTCCACGTCGTAAAGCTAAAATCATTCGTGATTACGGTAAACAGATGGCTGGTGATGATTGTGTTGCTTCTCGTCAGGATGAAGAT

**>PPP**

TGCAACACTAAAAAGCCAAACCTGGATGCTGAATTGGATCAACTGCTTCAGGGCCACTACATTAAAGGTTACCCAAAACAGTACACCTACTTCCTGGAAGACGGTAAAGTAAAAGTTTCTCGTCCAGAAGGTGTTAAAATTATTCCACCACAGTCTGATCGCCAGAAAATTGTTTTGCAGGCTCACAACCTGGCTCACACTGGTCGTGAGGCTACTCTGCTGAAAATTGCTAACCTGTACTGGTGGCCAAACATGCGTAAGGATGTTGTTAAACAGTTGGGTCGTTGCCAGCAATGCCTGATTACTAACGCATCTAACAAAGCATCTGGTCCAATTTTGCGTCCGGATCGTCCACAGAAACCATTCGATAAATTCTTCATTGATTACATTGGTCCGCTGCCTCCATCTCAGGGTTACCTGTACGTTTTGGTTGTAGTTGATGGTATGACCGGTTTCACTTGGCTGTACCCAACTAAAGCTCCATCCACTTCTGCAACTGTTAAGTCCTTGAACGTTCTGACCTCCATTGCTATTCCAAAAGTTATTCACTCTGATCAAGGCGCTGCTTTTACTTCTTCCACTTTCGCTGAATGGGCTAAAGAACGTGGTATTCACCTTGAATTTTCCACCCCATATCACCCGCAGTCTTCTGGTAAAGTTGAACGTAAAAACTCCGACATTAAACGTCTGCTGACTAAGCTGCTGGTAGGTCGTCCGACTAAATGGTACGATCTGTTACCGGTTGTTCAACTGGCTCTGAACAACACTTACTCTCCAGTACTGAAATACACTCCACACCAGCTCCTGTTCGGTATTGACTCTAACACTCCTTTCGCTAACCAGGATACTCTGGATCTGACTCGTGAAGAAGAACTGTCCTTGTTGCAGGAAATTCGTACCTCTCTGTACCACCCGTCTACTCCGCCAGCTTCTTCTCGTTCCTGGAGCCCAGTTGTTGGTCAGTTGGTTCAGGAACGTGTTGCTCGTCCTGCTTCTCTGCGCCCTCGTTGGCACAAACCATCTACTGTTCTGAAAGTTTTGAACCCACGCACCGTTGTTATTCTGGATCATCTGGGTAACAACCGTACTGTTTCCATTGATAACCTGAAACCAACCTCTCACCAGAACGGTACTACTAACGATACTGCTACTATGGATCACTTGGAAAAGAACGAA

**>TTT**

ACTATCACTCCAGAAACTTCTCGCCCGATTGATACCGAGTCTTGGAAATCTTACTACAAATCTGATCCACTGTGCTCTGCCGTTCTGATTCACATGAAAGAATTGACTCAGCACAACGTTACTCCGGAAGATATGTCTGCTTTCCGTTCTTACCAGAAGAAACTGGAACTGTCTGAAACTTTCCGCAAAAACTACTCTCTGGAAGATGAAATGATCTACTACCAGGATCGTCTGGTTGTTCCGATTAAACAGCAGAACGCAGTTATGCGCTTGTACCACGATCACACTCTGTTCGGCGGTCACTTCGGTGTTACTGTTACTCTGGCTAAGATTTCTCCAATTTACTACTGGCCAAAATTGCAGCACTCCATTATTCAGTACATTCGTACTTGCGTTCAGTGCCAACTGATTAAATCTCACCGTCCACGTCTTCACGGTCTGTTGCAGCCACTGCCAATTGCCGAAGGCCGTTGGCTGGACATTTCTATGGATTTCGTTACTGGTCTGCCACCAACTTCTAACAACCTGAACATGATCCTCGTTGTTGTTGATCGTTTCTCTAAACGTGCTCACTTCATTGCTACTCGTAAAACTTTGGATGCTACTCAACTGATCGATCTGCTGTTCCGTTACATTTTCTCTTACCACGGTTTCCCACGTACTATTACTTCTGATCGTGATGTTCGTATGACTGCTGATAAATACCAGGAACTGACTAAACGCCTGGGTATTAAATCCACTATGTCTTCTGCTAACCACCCACAGACTGATGGTCAGTCCGAACGTACCATTCAGACCCTGAACCGTTTGCTGCGTGCTTACGCTTCTACCAACATCCAGAACTGGCACGTTTACCTGCCACAGATTGAGTTTGTTTACAACTCCACTCCAACTCGTACCCTGGGTAAATCTCCATTCGAAATTGATCTGGGTTACTTGCCAAACACTCCAGCTATCAAGTCTGATGATGAAGTTAACGCTCGTTCTTTCACTGCTGTAGAATTGGCTAAGCACCTGAAGGCTCTGACTATTCAGACTAAAGAGCAGCTTGAACACGCTCAGATTGAAATGGAAACTAACAACAACCAGCGTCGCAAACCACTGCTGCTGAACATTGGTGATCACGTACTGGTTCACCGTGATGCTTACTTCAAAAAGGGTGCTTATATGAAAGTTCAGCAAATTTACGTTGGTCCATTCCGTGTAGTTAAGAAAATTAACGATAACGCTTACGAACTGGATTTGAACTCCCACAAAAAGAAACACCGTGTTATTAACGTTCAGTTCTTGAAAAAGTTCGTTTACCGTCCTGATGCATACCCGAAAAACAAACCAATTTCTTCTACGGAACGTATTAAGCGTGCACACGAAGTTACTGCTCTGATTGGTATTGATACTACTCACAAAACTTACCTGTGCCACATGCAAGATGTTGATCCAACTCTGTCTGTTGAATACTCTGAAGCAGAATTTTGCCAGATTCCTGAACGTACTCGTCGTTCTATCCTGGCTAATTTCCGTCAGTTGTACGAAACTCAGGACAACCCAGAACGTGAGGAAGATGTAGTTTCTCAGAACGAAATTTGCCAGTACGATAACACTTCCCCA
