# Supplementary material for: Directed DNA Shuffling of Retrovirus and Retrotransposon Integrase Protein Domains
Source: PLoS One. 2013 May 17;8(5):e63957. doi: 10.1371/journal.pone.0063957 (PMC3656877; doi:10.1371/journal.pone.0063957)
Supplement: Table S3 — Oligonucleotides for integrase gene assembly. (DOCX) [file pone.0063957.s004.docx]

**Table S3. Oligonucleotides for integrase gene assembly.**

| **Name** | **Oligonucleotide Sequence** |
| --- | --- |
| On1 fwd (Pf1-1) | ACTATCACTCCAGAAACTTCTCGCCCGATTGATACCGAGTCTTGGAAATCTTACTACAAA |
| On2 rev (Pf1-2) | GAACGGCAGAGCACAGTGGATCAGATTTGTAGTAAGATTTCCAAGACTCGGTATCAATCG |
| On3 fwd (Pf1-3) | TCTGATCCACTGTGCTCTGCCGTTCTGATTCACATGAAAGAATTGACTCAGCACAAC |
| On4 rev (Pf1-4) | GGAAAGCAGACATATCTTCCGGAGTAACGTTGTGCTGAGTCAATTCTTTCATGTGAATCA |
| On5 fwd (Pf1-5) | GTTACTCCGGAAGATATGTCTGCTTTCCGTTCTTACCAGAAGAAACTGGAACTGTCTGAA |
| On6 rev (Pf1-6) | CTTCCAGAGAGTAGTTTTTGCGGAAAGTTTCAGACAGTTCCAGTTTCTTCTGGTAAGAAC |
| On7 fwd (Pf1-7) | ACTTTCCGCAAAAACTACTCTCTGGAAGATGAAATGATCTACTACCAGGATCGTCTGGTT |
| On8 rev (Pf1-8) | TAACTGCGTTCTGCTGTTTAATCGGAACAACCAGACGATCCTGGTAGTAGATCATTTCAT |
| On9 fwd (Pf1-9 Pf2-1) | GTTCCGATTAAACAGCAGAACGCAGTTATGCGCTTGTACCACGATCACACTCTGTTCGGC |
| On10 rev (Pf1-10 Pf2-2) | CAGAGTAACAGTAACACCGAAGTGACCGCCGAACAGAGTGTGATCGTGGTACAAGCGCA |
| On11 fwd (Pf2-3) | GGTCACTTCGGTGTTACTGTTACTCTGGCTAAGATTTCTCCAATTTACTACTGGCCA |
| On12 rev (Pf2-4) | AATGTACTGAATAATGGAGTGCTGCAATTTTGGCCAGTAGTAAATTGGAGAAATCTTAGC |
| On13 fwd (Pf2-5) | AAATTGCAGCACTCCATTATTCAGTACATTCGTACTTGCGTTCAGTGCCAACTGATTAAA |
| On14 rev (Pf2-6) | CAACAGACCGTGAAGACGTGGACGGTGAGATTTAATCAGTTGGCACTGAACGCAAGTACG |
| On15 fwd (Pf2-7) | TCTCACCGTCCACGTCTTCACGGTCTGTTGCAGCCACTGCCAATTGCCGAAGGCCGTTGG |
| On16 rev (Pf2-8) | ACCAGTAACGAAATCCATAGAAATGTCCAGCCAACGGCCTTCGGCAATTGGCAGTGGCTG |
| On17 fwd (Pf2-9 Pf3-1) | CTGGACATTTCTATGGATTTCGTTACTGGTCTGCCACCAACTTCTAACAACCTGAACATG |
| On18 rev (Pf2-10 Pf3-2) | AGAGAAACGATCAACAACAACGAGGATCATGTTCAGGTTGTTAGAAGTTGGTGGCAG |
| On19 fwd (Pf3-3) | ATCCTCGTTGTTGTTGATCGTTTCTCTAAACGTGCTCACTTCATTGCTACTCGTAAA |
| On20 rev (Pf3-4) | CAGATCGATCAGTTGAGTAGCATCCAAAGTTTTACGAGTAGCAATGAAGTGAGCACGTTT |
| On21 fwd (Pf3-5) | ACTTTGGATGCTACTCAACTGATCGATCTGCTGTTCCGTTACATTTTCTCTTACCACGGT |
| On22 rev (Pf3-6) | ATCACGATCAGAAGTAATAGTACGTGGGAAACCGTGGTAAGAGAAAATGTAACGGAACAG |
| On23 fwd (Pf3-7) | TTCCCACGTACTATTACTTCTGATCGTGATGTTCGTATGACTGCTGATAAATACCAGGAA |
| On24 rev (Pf3-8) | AGTGGATTTAATACCCAGGCGTTTAGTCAGTTCCTGGTATTTATCAGCAGTCATACGAAC |
| On25 fwd (Pf3-9 Pf4-1) | CTGACTAAACGCCTGGGTATTAAATCCACTATGTCTTCTGCTAACCACCCACAGACTGAT |
| On26 rev (Pf3-10 Pf4-2) | CAGGGTCTGAATGGTACGTTCGGACTGACCATCAGTCTGTGGGTGGTTAGCAGAAGACAT |
| On27 fwd (Pf4-3) | GGTCAGTCCGAACGTACCATTCAGACCCTGAACCGTTTGCTGCGTGCTTACGCTTCTACC |
| On28 rev (Pf4-4) | TGGCAGGTAAACGTGCCAGTTCTGGATGTTGGTAGAAGCGTAAGCACGCAGCAAACGGTT |
| On29 fwd (Pf4-5) | AACATCCAGAACTGGCACGTTTACCTGCCACAGATTGAGTTTGTTTACAACTCCACTCCA |
| On30 rev (Pf4-6) | TTCGAATGGAGATTTACCCAGGGTACGAGTTGGAGTGGAGTTGTAAACAAACTCAATCTG |
| On31 fwd (Pf4-7) | ACTCGTACCCTGGGTAAATCTCCATTCGAAATTGATCTGGGTTACTTGCCAAACACTCCA |
| On32 rev (Pf4-8) | AGCGTTAACTTCATCATCAGACTTGATAGCTGGAGTGTTTGGCAAGTAACCCAGATCAAT |
| On33 fwd (Pf4-9 Pf5-1) | GCTATCAAGTCTGATGATGAAGTTAACGCTCGTTCTTTCACTGCTGTAGAATTGGCTA |
| On34 rev (Pf4-10 Pf5-2) | CTGAATAGTCAGAGCCTTCAGGTGCTTAGCCAATTCTACAGCAGTGAAAGAACG |
| On35 fwd (Pf5-3) | AGCACCTGAAGGCTCTGACTATTCAGACTAAAGAGCAGCTTGAACACGCTCAGATTGAA |
| On36 rev (Pf5-4) | GCGACGCTGGTTGTTGTTAGTTTCCATTTCAATCTGAGCGTGTTCAAGCTGCTCTTTAGT |
| On37 fwd (Pf5-5) | ATGGAAACTAACAACAACCAGCGTCGCAAACCACTGCTGCTGAACATTGGTGATCACGTA |
| On38 rev (Pf5-6) | TTTGAAGTAAGCATCACGGTGAACCAGTACGTGATCACCAATGTTCAGCAGCAGTGGTTT |
| On39 fwd (Pf5-7) | CTGGTTCACCGTGATGCTTACTTCAAAAAGGGTGCTTATATGAAAGTTCAGCAAATTTAC |
| On40 rev (Pf5-8) | TTTCTTAACTACACGGAATGGACCAACGTAAATTTGCTGAACTTTCATATAAGCACCCTT |
| On41 fwd (Pf5-9 Pf6-1) | GTTGGTCCATTCCGTGTAGTTAAGAAAATTAACGATAACGCTTACGAACTGGATTTGA |
| On42 rev (Pf5-10 Pf6-2) | AACACGGTGTTTCTTTTTGTGGGAGTTCAAATCCAGTTCGTAAGCGTTATCGTTAAT |
| On43 fwd (Pf6-3) | ACTCCCACAAAAAGAAACACCGTGTTATTAACGTTCAGTTCTTGAAAAAGTTCGTTTAC |
| On44 rev (Pf6-4) | TTTGTTTTTCGGGTATGCATCAGGACGGTAAACGAACTTTTTCAAGAACTGAACGTTAAT |
| On45 fwd (Pf6-5) | CGTCCTGATGCATACCCGAAAAACAAACCAATTTCTTCTACGGAACGTATTAAGCGTGCA |
| On46 rev (Pf6-6) | AATACCAATCAGAGCAGTAACTTCGTGTGCACGCTTAATACGTTCCGTAGAAGAAATTGG |
| On47 fwd (Pf6-7) | CACGAAGTTACTGCTCTGATTGGTATTGATACTACTCACAAAACTTACCTGTGCCACATG |
| On48 rev (Pf6-8) | AACAGACAGAGTTGGATCAACATCTTGCATGTGGCACAGGTAAGTTTTGTGAGTAGTATC |
| On49 fwd (Pf6-9 Pf7-1) | CAAGATGTTGATCCAACTCTGTCTGTTGAATACTCTGAAGCAGAATTTTGCCAGATTC |
| On50 rev (Pf6-10 Pf7-2) | CAGGATAGAACGACGAGTACGTTCAGGAATCTGGCAAAATTCTGCTTCAGAGTATTC |
| On51 fwd (Pf7-3) | CTGAACGTACTCGTCGTTCTATCCTGGCTAATTTCCGTCAGTTGTACGAAACTCAGGAC |
| On52 rev (Pf7-4) | AACTACATCTTCCTCACGTTCTGGGTTGTCCTGAGTTTCGTACAACTGACGGAAATTAGC |
| On53 fwd (Pf7-5) | AACCCAGAACGTGAGGAAGATGTAGTTTCTCAGAACGAAATTTGCCAGTACGATAACACT |
| On54 rev (Pf7-6) | TTTATCGATGCCATCCAGGAATGGGGAAGTGTTATCGTACTGGCAAATTTCGTTCTGAGA |
| On55 fwd (Pf7-7) | TCCCCATTCCTGGATGGCATCGATAAAGCTCAGGAAGAACACGAAAAATACCACTCTAAC |
| On56 rev (Pf7-8) | GTTGAAATCAGAAGCCATTGCACGCCAGTTAGAGTGGTATTTTTCGTGTTCTTCCTGAGC |
| On57 fwd (Pf7-9 Pf8-1) | TGGCGTGCAATGGCTTCTGATTTCAACTTGCCACCTGTTGTTGCAAAAGAAATCGTTG |
| On58 rev (Pf7-10 Pf8-2) | CTTCAGTTGACATTTGTCGCAGGAAGCAACGATTTCTTTTGCAACAACAGGTGGCAA |
| On59 fwd (Pf8-3) | CTTCCTGCGACAAATGTCAACTGAAGGGTGAAGCTATGCACGGTCAGGTTGATTGCTCT |
| On60 rev (Pf8-4) | AGTGCAATCCAACTGCCAAATACCTGGAGAGCAATCAACCTGACCGTGCATAGCTTCACC |
| On61 fwd (Pf8-5) | CCAGGTATTTGGCAGTTGGATTGCACTCACCTGGAAGGGAAAGTAATTCTGGTTGCTGTT |
| On62 rev (Pf8-6) | AGCTTCAATGTAACCAGAAGCTACGTGAACAGCAACCAGAATTACTTTCCCTTCCAGGTG |
| On63 fwd (Pf8-7) | CACGTAGCTTCTGGTTACATTGAAGCTGAGGTTATTCCGGCTGAAACTGGTCAGGAAACT |
| On64 rev (Pf8-8) | ACCTGCCAATTTCAGCAAGAAATAAGCAGTTTCCTGACCAGTTTCAGCCGGAATAACCTC |
| On65 fwd (Pf8-9 Pf9-1) | GCTTATTTCTTGCTGAAATTGGCAGGTCGCTGGCCGGTTAAAACTGTTCACACTGATAA |
| On66 rev (Pf8-10 Pf9-2) | AACAGTAGTAGAAGTGAAGTTGGAACCGTTATCAGTGTGAACAGTTTTAACCGGCCAGCG |
| On67 fwd (Pf9-3) | CGGTTCCAACTTCACTTCTACTACTGTTAAAGCTGCATGTTGGTGGGCTGGTATCAAA |
| On68 rev (Pf9-4) | CTGTGGGTTGTATGGAATACCAAATTCCTGTTTGATACCAGCCCACCAACATGCAGCTTT |
| On69 fwd (Pf9-5) | CAGGAATTTGGTATTCCATACAACCCACAGTCCCAAGGCGTTATTGAATCTATGAACAAA |
| On70 rev (Pf9-6) | ACGAACTTGACCAATAATCTTTTTCAGCTCTTTGTTCATAGATTCAATAACGCCTTGGGA |
| On71 fwd (Pf9-7) | GAGCTGAAAAAGATTATTGGTCAAGTTCGTGATCAGGCTGAACACCTGAAAACTGCTGTG |
| On72 rev (Pf9-8) | TTTGAAGTTGTGAATGAATACTGCCATCTGCACAGCAGTTTTCAGGTGTTCAGCCTGATC |
| On73 fwd (Pf9-9 Pf10-1) | CAGATGGCAGTATTCATTCACAACTTCAAACGTAAAGGTGGTATTGGTGGTTACAGCGCA |
| On74 rev (Pf9-10 Pf10-2) | AGTTGCAATAATATCAACAATGCGTTCACCTGCGCTGTAACCACCAATACCACCTTTACG |
| On75 fwd (Pf10-3) | GGTGAACGCATTGTTGATATTATTGCAACTGACATTCAAACCAAGGAATTGCAGAAACAG |
| On76 rev (Pf10-4) | GTAAACACGGAAGTTCTGAATCTTGGTAATCTGTTTCTGCAATTCCTTGGTTTGAATGTC |
| On77 fwd (Pf10-5) | ATTACCAAGATTCAGAACTTCCGTGTTTACTACCGTGATTCTCGTGATCCAGTTTGGAAA |
| On78 rev (Pf10-6) | TTCACCTTTCCACAACAGTTTAGCTGGGCCTTTCCAAACTGGATCACGAGAATCACGGTA |
| On79 fwd (Pf10-7) | GGCCCAGCTAAACTGTTGTGGAAAGGTGAAGGTGCTGTTGTTATCCAGGATAACTCTGAT |
| On80 rev (Pf10-8) | TTTAGCTTTACGACGTGGAACAACTTTAATATCAGAGTTATCCTGGATAACAACAGCACC |
| On81 fwd (Pf10-9 Pf11-1) | ATTAAAGTTGTTCCACGTCGTAAAGCTAAAATCATTCGTGATTACGGTAAACAGATGGCT |
| On82 rev (Pf10-10 Pf11-2) | CTGACGAGAAGCAACACAATCATCACCAGCCATCTGTTTACCGTAATCACGAATGAT |
| On83 fwd (Pf11-3) | GGTGATGATTGTGTTGCTTCTCGTCAGGATGAAGATGGTCCAGGTTGCAACACTAAAAAG |
| On84 rev (Pf11-4) | TTGATCCAATTCAGCATCCAGGTTTGGCTTTTTAGTGTTGCAACCTGGACCATCTTCATC |
| On85 fwd (Pf11-5) | CCAAACCTGGATGCTGAATTGGATCAACTGCTTCAGGGCCACTACATTAAAGGTTACCCA |
| On86 rev (Pf11-6) | GTCTTCCAGGAAGTAGGTGTACTGTTTTGGGTAACCTTTAATGTAGTGGCCCTGAAGCAG |
| On87 fwd (Pf11-7) | AAACAGTACACCTACTTCCTGGAAGACGGTAAAGTAAAAGTTTCTCGTCCAGAAGGTGTT |
| On88 rev (Pf11-8) | GCGATCAGACTGTGGTGGAATAATTTTAACACCTTCTGGACGAGAAACTTTTACTTTACC |
| On89 fwd (Pf11-9 Pf12-1) | AAAATTATTCCACCACAGTCTGATCGCCAGAAAATTGTTTTGCAGGCTCACAACCTGGCT |
| On90 rev (Pf11-10 Pf12-2) | CAGCAGAGTAGCCTCACGACCAGTGTGAGCCAGGTTGTGAGCCTGCAAAACAATTTTCTG |
| On91 fwd (Pf12-3) | CACACTGGTCGTGAGGCTACTCTGCTGAAAATTGCTAACCTGTACTGGTGGCCAAACATG |
| On92 rev (Pf12-4) | ACCCAACTGTTTAACAACATCCTTACGCATGTTTGGCCACCAGTACAGGTTAGCAATTTT |
| On93 fwd (Pf12-5) | CGTAAGGATGTTGTTAAACAGTTGGGTCGTTGCCAGCAATGCCTGATTACTAACGCATCT |
| On94 rev (Pf12-6) | ACGCAAAATTGGACCAGATGCTTTGTTAGATGCGTTAGTAATCAGGCATTGCTGGCAACG |
| On95 fwd (Pf12-7) | AACAAAGCATCTGGTCCAATTTTGCGTCCGGATCGTCCACAGAAACCATTCGATAAATTC |
| On96 rev (Pf12-8) | AGGCAGCGGACCAATGTAATCAATGAAGAATTTATCGAATGGTTTCTGTGGACGATCCGG |
| On97 fwd (Pf12-9 Pf13-1) | TTCATTGATTACATTGGTCCGCTGCCTCCATCTCAGGGTTACCTGTACGTTTTGGTTG |
| On98 rev (Pf12-10 Pf13-2) | AGTGAAACCGGTCATACCATCAACTACAACCAAAACGTACAGGTAACCCTGAGATGG |
| On99 fwd (Pf13-3) | TAGTTGATGGTATGACCGGTTTCACTTGGCTGTACCCAACTAAAGCTCCATCCACTTCT |
| On100 rev (Pf13-4) | CAGAACGTTCAAGGACTTAACAGTTGCAGAAGTGGATGGAGCTTTAGTTGGGTACAGCCA |
| On101 fwd (Pf13-5) | GCAACTGTTAAGTCCTTGAACGTTCTGACCTCCATTGCTATTCCAAAAGTTATTCACTCT |
| On102 rev (Pf13-6) | GGAAGAAGTAAAAGCAGCGCCTTGATCAGAGTGAATAACTTTTGGAATAGCAATGGAGGT |
| On103 fwd (Pf13-7) | GATCAAGGCGCTGCTTTTACTTCTTCCACTTTCGCTGAATGGGCTAAAGAACGTGGTATT |
| On104 rev (Pf13-8) | GTGATATGGGGTGGAAAATTCAAGGTGAATACCACGTTCTTTAGCCCATTCAGCGAAAGT |
| On105 fwd (Pf13-9 Pf14-1) | CACCTTGAATTTTCCACCCCATATCACCCGCAGTCTTCTGGTAAAGTTGAACGTAAAAAC |
| On106 rev (Pf13-10 Pf14-2) | CTTAGTCAGCAGACGTTTAATGTCGGAGTTTTTACGTTCAACTTTACCAGAAGACTGCGG |
| On107 fwd (Pf14-3) | TCCGACATTAAACGTCTGCTGACTAAGCTGCTGGTAGGTCGTCCGACTAAATGGTACGAT |
| On108 rev (Pf14-4) | CAGAGCCAGTTGAACAACCGGTAACAGATCGTACCATTTAGTCGGACGACCTACCAGCAG |
| On109 fwd (Pf14-5) | CTGTTACCGGTTGTTCAACTGGCTCTGAACAACACTTACTCTCCAGTACTGAAATACACT |
| On110 rev (Pf14-6) | GTCAATACCGAACAGGAGCTGGTGTGGAGTGTATTTCAGTACTGGAGAGTAAGTGTTGTT |
| On111 fwd (Pf14-7) | CCACACCAGCTCCTGTTCGGTATTGACTCTAACACTCCTTTCGCTAACCAGGATACTCTG |
| On112 rev (Pf14-8) | GGACAGTTCTTCTTCACGAGTCAGATCCAGAGTATCCTGGTTAGCGAAAGGAGTGTTAGA |
| On113 fwd (Pf14-9 Pf15-1) | GATCTGACTCGTGAAGAAGAACTGTCCTTGTTGCAGGAAATTCGTACCTCTCTGTACC |
| On114 rev (Pf14-10 Pf15-2) | AGAAGAAGCTGGCGGAGTAGACGGGTGGTACAGAGAGGTACGAATTTCCTGCAACAA |
| On115 fwd (Pf15-3) | ACCCGTCTACTCCGCCAGCTTCTTCTCGTTCCTGGAGCCCAGTTGTTGGTCAGTTG |
| On116 rev (Pf15-4) | AGAAGCAGGACGAGCAACACGTTCCTGAACCAACTGACCAACAACTGGGCTCCAGGAACG |
| On117 fwd (Pf15-5) | GTTCAGGAACGTGTTGCTCGTCCTGCTTCTCTGCGCCCTCGTTGGCACAAACCATCTACT |
| On118 rev (Pf15-6) | AACGGTGCGTGGGTTCAAAACTTTCAGAACAGTAGATGGTTTGTGCCAACGAGGGCGCAG |
| On119 fwd (Pf15-7) | GTTCTGAAAGTTTTGAACCCACGCACCGTTGTTATTCTGGATCATCTGGGTAACAACCGT |
| On120 rev (Pf15-8) | GGTTGGTTTCAGGTTATCAATGGAAACAGTACGGTTGTTACCCAGATGATCCAGAATAAC |
| On121 fwd (Pf15-9) | ACTGTTTCCATTGATAACCTGAAACCAACCTCTCACCAGAACGGTACTACTAACGA |
| On122 rev (Pf15-10) | TTCGTTCTTTTCCAAGTGATCCATAGTAGCAGTATCGTTAGTAGTACCGTTCTGGTGAGA |
