# Supplementary material for: Directed DNA Shuffling of Retrovirus and Retrotransposon Integrase Protein Domains
Source: PLoS One. 2013 May 17;8(5):e63957. doi: 10.1371/journal.pone.0063957 (PMC3656877; doi:10.1371/journal.pone.0063957)
Supplement: Table S4 — Oligonucleotides used in crossover PCR. (DOCX) [file pone.0063957.s005.docx]

**Table S4. Oligonucleotides used in crossover PCR.**

| **Oligo** | **Sequence (5’-3’)** |
| --- | --- |
| HPX | GGTGAAGCTATGCACGGTCAGGTTGATTGCGATCGTCCACAGAAACCATTCGATAAATTC |
| HTX | GGTGAAGCTATGCACGGTCAGGTTGATTGCCCAATTGCCGAAGGCCGTTGGCTGGACAT |
| PHX | TAACAAAGCATCTGGTCCAATTTTGCGTCCGTCTCCAGGTATTTGGCAGTTGGATTGC |
| PTX | CTGTTCGGTATTGACTCTAACACTCCTTTCATCAAGTCTGATGATGAAGTTAACGCTCGT |
| THX | CCACGTCTTCACGGTCTGTTGCAGCCACTGTCTCCAGGTATTTGGCAGTTGGATTGCACT |
| TPX | CCACGTCTTCACGGTCTGTTGCAGCCACTGGATCGTCCACAGAAACCATTCGATAAATTC |
| XHP | CCAAGGAATTGCAGAAACAGATTACCAAGATTGCTAACCAGGATACTCTGGATCTGACTC |
| XHT | CAAGGAATTGCAGAAACAGATTACCAAGATTATCAAGTCTGATGATGAAGTTAACGCTCG |
| XPH | CCTGTTCGGTATTGACTCTAACACTCCTTTCCAGAACTTCCGTGTTTACTACCGTGATTC |
| XPT | CTGTTCGGTATTGACTCTAACACTCCTTTCATCAAGTCTGATGATGAAGTTAACGCTCGT |
| XTH | GATCTGGGTTACTTGCCAAACACTCCAGCTCAGAACTTCCGTGTTTACTACCGTGATTC |
| XTP | GATCTGGGTTACTTGCCAAACACTCCAGCTGCTAACCAGGATACTCTGGATCTGACTCGT |
