# Supplementary material for: Directed DNA Shuffling of Retrovirus and Retrotransposon Integrase Protein Domains
Source: PLoS One. 2013 May 17;8(5):e63957. doi: 10.1371/journal.pone.0063957 (PMC3656877; doi:10.1371/journal.pone.0063957)
Supplement: Table S5 — Oligonucleotides used for in vitro strand-transfer assay. (DOCX) [file pone.0063957.s006.docx]

**Table S5. Oligonucleotides used for *in vitro* strand-transfer assay.**

| **Oligo** | **Sequence** | **Description** |
| --- | --- | --- |
| 242 | GGAACTGCTGATCATCTCT | Reverse primer, anneals to pLY1855 (*SNR6* at +67) |
| HH1707 | GCATTGGCTCAAAGGTCCAAACC | Primer, anneals to substrate DNA |
| 679 | ACTCCCCGTCGTGTAGATAACTACG | Forward primer, control PCR |
| 680 | AAGCCATACCAAACGACGAGC | Reverse primer, control PCR |
| HH1704 | GCATTGGCTCAAAGGTCCAAACCctcgagcccgtaatacaaca | Plus-strand oligo for substrate mimicking pre-processed Ty3 U5 LTR terminus, underlined region is same as HH1707 |
| HH1706 | ggtgttgtattacgggctcgagGGTTTGGACCTTTGAGCCAATGC | Minus-strand oligo for substrate mimicking Ty3 U5 LTR terminus |
| XQ3357 | GCATTGGCTCAAAGGTCCAAACCtatacaaaattccatgaca | Plus-strand oligo for substrate mimicking pre-processed PFV U5 LTR terminus |
| XQ3359 | attgtcatggaattttgtataGGTTTGGACCTTTGAGCCAATGC | Minus-strand oligo for substrate mimicking PFV U5 LTR terminus |
| XQ3360 | GCATTGGCTCAAAGGTCCAAACCatgtggaaaatctctagca | Plus-strand oligo for substrate mimicking pre-processed HIV U5 LTR terminus |
| XQ3362 | actgctagagattttccacatGGTTTGGACCTTTGAGCCAATGC | Minus-strand oligo for substrate mimicking HIV U5 LTR terminus |
